# The use of an electronic medication organizer device with alarm to improve medication adherence of older adults with hypertension

**DOI:** 10.31744/einstein_journal/2021AO6011

**Published:** 2021-09-13

**Authors:** Liliana Batista Vieira, Adriano Max Moreira Reis, Celso de Ávila Ramos, Tiago Marques dos Reis, Silvia Helena de Bortoli Cassiani

**Affiliations:** 1 Universidade de São Paulo Escola de Enfermagem de Ribeirão Preto Ribeirão PretoSP Brazil Escola de Enfermagem de Ribeirão Preto, Universidade de São Paulo, Ribeirão Preto, SP, Brazil.; 2 Universidade Federal de Minas Gerais Faculdade de Farmácia Belo HorizonteMG Brazil Faculdade de Farmácia, Universidade Federal de Minas Gerais, Belo Horizonte, MG, Brazil.; 3 Universidade José do Rosário Vellano AlfenasMG Brazil Universidade José do Rosário Vellano, Alfenas, MG, Brazil.; 4 Universidade Federal de Alfenas AlfenasMG Brazil Universidade Federal de Alfenas, Alfenas, MG, Brazil.

**Keywords:** Drug utilization, Equipment and supplies, Reminder systems, Aged, Patient safety

## Abstract

**Objective:**

To examine whether the use of a monthly electronic medication organizer device equipped with an alarm clock, called Electronic System for Personal and Controlled Use of Medications (Supermed), improves medication adherence of older adults with hypertension.

**Methods:**

This is a quali-quantitative, prospective, before-and-after study performed with 32 older adult patients with diagnosis of hypertension, who were recruited at a Primary Care Unit in Brazil.

**Results:**

The main outcome measures were improvement of medication adherence and blood pressure control after intervention with Supermed. Regarding medication adherence, 81.2% of patients were “less adherent” in the pre-intervention period, and 96.9% were “more adherent” in the post-intervention period. This means that 78.1% of patients changed from “less adherent” to “more adherent” after the intervention with Supermed (p<0.001). The mean systolic and diastolic blood pressure differences between intervention day and post-intervention were 18.5mmHg (p<0.0001) and 4.3mmHg (p<0.007), respectively, and the differences between mean systolic and diastolic blood pressure between pre-intervention and post-intervention were 21.6mmHg (p<0.001) and 4.7mmHg (p<0.001) respectively.

**Conclusion:**

The use of Supermed significantly improved self-reported medication adherence and blood pressure control in a hypertensive older adult population.

## INTRODUCTION

Hypertension is a major modifiable risk factor for cardiovascular events and mortality in older people.^(^^1^^)^ Despite the availability of effective antihypertensive drug therapy, the lack of adherence to treatment may compromise blood pressure control,^(^^1^^–^^4^^)^ and the use of evidence-based drug therapy decreases cardiovascular morbidity and mortality.^(^^1^^,^^2^^)^

Adherence to drug therapy is a complex and individual psychobehavioral phenomenon. It is an increasing problem among older adults influenced by factors associated with the physician, specific treatment, healthcare system, comorbidities, patient's understanding of their medical condition, and patient's satisfaction with treatment.^(^^4^^,^^5^^)^ This variety of factors can lead to intentional or unintentional patient non-adherence.^(^^4^^)^ Intentional non-adherence is determined by the patients’ active decision of not taking their medication as prescribed. Alternatively, unintentional non-adherence results from other factors such as forgetfulness, misunderstanding of the medication regimens, and lack of access to medications.^(^^4^^,^^6^^)^ Strategies that remind patients about medication intake are important to improve adherence.^(^^3^^)^

Pharmacists are some of the most important professionals to promote the rational use of medications, since they ensure that drug regimens are safe and effective. The strategies that clinical pharmacists may adopt to improve medication adherence include providing instructions to patients about their disease, treatment, and lifestyle; monitoring and recording blood pressure; providing medication reminders, including phone calls; and furnishing dispensing systems, such as electronic monitoring, blisters or pillboxes to organize daily doses.^(^^7^^–^^9^^)^ In Brazil, there are some daily and weekly medicine box organizers, equipped with alarm clocks, but not an electronic monthly organizer device.

One monthly custom medication device, named Electronic System for Personal and Controlled Use of Medications, or Supermed, was developed.^(^^10^^)^ The device is equipped with an alarm clock and helps older adults to organize and manage their daily intake of medicines for 1 month, in a practical and easy way, and aids the health professionals in assessing whether the patients were taking the medicines correctly. Supermed offers to older adults, who are usually not familiar with modern technology, clear, simple, and direct instructions; its use requires minimum physical effort and knowledge about electronic devices.^(^^10^^)^ The hypothesis tested in the study was that the use of Supermed may increase patient adherence to medication treatment.

## OBJECTIVE

To evaluate whether the use of Electronic System for Personal and Controlled Use of Medications improves medication adherence in older adults with hypertension.

## METHODS

### Study design and setting

The research was carried out with two methodological approaches, quantitative and qualitative, in a complementary way for a more in-depth understanding of the studied phenomena. The quantitative stage was performed with a quasi-experimental, prospective, before-and-after design. In turn, the qualitative stage was carried out with thematic content analysis. Both stage were developed at a Primary Care Unit in the city of Ribeirão Preto (SP), Brazil.

### Population

The convenience sample consisted of 32 older adult patients, considering the selection criteria and the research schedule. The inclusion criteria were age ≥60 years; both genders; able to speak Portuguese; daily intake of five or more solid drugs, including at least one antihypertensive agent; systolic blood pressure (SBP) ≥140mmHg, or SBP ≥130mmHg, if hypertensive and diabetic; regular following by a physician from the Primary Care Unit. The SBP values were defined according to the Brazilian Hypertension Guidelines.^(^^11^^)^ The exclusion criteria were older adult diagnosed with mental disorders or cognitive impairment. The inclusion criterion of five or more drugs aimed to ensure the participation of the older adults with major polypharmacy (more than five medications per patient).^(^^12^^)^

### Ethical approval

All the older adults who accepted to participate in this study signed the Informed Consent Form. All the procedures performed in studies involving human participants complied with the ethical standards of the institutional Research Ethics Committee and with the 1964 Helsinki declaration and its later amendments, or comparable ethical standards (Research Ethics Committee of the *Universidade de São Paulo* , Ribeirão Preto, São Paulo, Brazil College of Nursing, protocol 1,398/2011).

### Intervention

**Figure 1:**
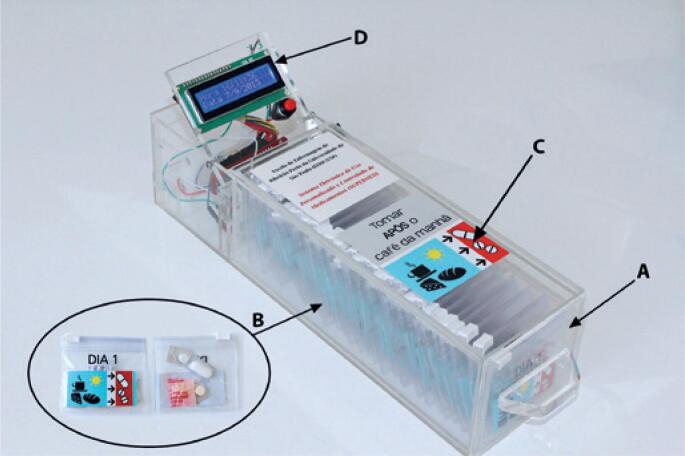
Electronic System for Personal and Controlled Use of Medications, a monthly electronic medication organizer device with an alarm clock. A) Medication organizer device identified with the correct intake time, and filled with sachets containing a single dose of the prescribed medications, in amounts sufficient for one month of treatment. The names of the medications are written on a label at the back of each device; B) Each sachet containing a single dose is identified with the calendar day and intake time; C) Details of the labels: “take after breakfast”; D) Alarm programmed to ring in the time agreed with the older adult

The Supermed was used as the medication reminder device. It is composed of a medication organizer device, plastic compartments to place individual doses of the medications, instruction labels, an alarm clock, and an electronic system that controls the alarm clock and records the box opening ( Figure 1 ).^(^^10^^)^

The Supermed was designed to remind the patient about the time to take the medication; check whether the patient turned-off the alarm clock and opened the box to take the medication; record the times at which the device was opened, which presupposes medication intake. The Supermed prototype includes a drawer with capacity of one month's supply of medicines, sound and visual medication timers, and a memory card for recording the times when the box was opened/closed (scheduled and unscheduled). This information is later transferred to a computer. Evolutionary prototyping was used to develop Supermed with the Arduino platform and C programming. To read alarm and box opening/closing data, software was developed in the Java language.^(^^10^^)^

The total number of devices used varied among the patients. Each Supermed had a different colour, and was filled with compartments (sachets) containing single doses of the prescribed medications. They received devices identified with the medication intake time, for example: “after waking up”, “before breakfast”, “after breakfast”, “before lunch”, “after lunch”, “before dinner”, “after dinner” and/or “at bedtime”. The names of the medications contained in each box were written on a label at the back, and the total number of compartments was sufficient for one month of treatment. The boxes were prepared monthly in the presence of the patient, as an opportunity to clarify their doubts, and improve their understanding of their medical condition, the medication instructions, and the importance of adherence to treatment. This meeting lasted for 1 to 2 hours for each patient. The patients received a bag to transport the boxes, and after the end of the study period, they were allowed to keep the devices.

The Supermed was introduced to the Primary Care Unit in February 2013. The pre-intervention period comprised data from patients between March 2012 and January 2013, and the post-intervention period, data collected between February and August 2013, from the same patients. This article presents tests and results of functionality of the device alarm. Others functionalities of Supermed, such as the record of times, will be presented in the near future.

### Outcomes and measures

#### Blood pressure

Blood pressure was measured as recommended by the Brazilian Hypertension Guidelines,^(^^11^^)^ using a calibrated and validated digital blood pressure monitor (model Omron HEM-742INT). Each measurement was performed in triplicate, and the mean value from the two last measurements was calculated. Systolic blood pressure and diastolic blood pressure (DBP) measurements were performed at the following time points:

– Pre-intervention period: before introduction of Supermed, five meetings were held with each patient, from March 2012 to January 2013.
– Intervention day: on the day of Supermed introduction, one meeting with patient adult was held during February 2013.
– Post-intervention period: after introduction of Supermed, five meetings (one meeting per month) were held with each patient, from March 2013 to August 2013.

#### Medication adherence

It was determined using the Morisky Medication Adherence Scale,^(^^13^^)^ which was composed of four questions, to analyse the patients’ habitual behaviours with respect to the medicine intake, in the pre- and post-intervention periods. The answers were scored as yes (zero) and no (one); patients with final scores of zero to two and three to four were considered as “less adherent” and “more adherent”, respectively. The test was applied before and after the intervention.

#### Satisfaction survey

At the end of the study period (August 2013), a semi-structured interview was conducted with each patient, using four guiding questions: What do you think about the medicine box organizers? What do you think about the alarm clock? What do you think about the instructions to use Supermed, such as the illustrated labels? How do you evaluate your general health in the last few months? The interviews were recorded and fully transcribed. Data were analysed through thematic content analysis following the editorial analysis style. The content analysis aims to deconstruct the speech to see “what lies behind the manifested contents”.^(^^14^^)^

### Data analysis

Data were analysed using SAS^®^ 9.0 statistical software. The variables of interest (SBP and DBP) in the three data collection periods (pre-intervention, intervention day, and post-intervention) were compared using analysis of variance (Anova) and *t* -based orthogonal contrasts. The variables from the Morisky scale^(^^13^^)^ obtained before and after the intervention were compared using the McNemar's test. Values of p<0.05 were considered significant.

## RESULTS

This study included 32 older adult patients (21 women), with mean age of 71.4, standard deviation (SD) of 5.6 years. Most of whom were retired (87.5%) and had low incomes – below three minimum wages – (62.6%) and low educational levels – illiterate or less than 4 years of school study – (78.2%). The patients had been diagnosed with hypertension for an average of 19.4 years before study enrolment, and had been taking long-term medicines since then to treat not only hypertension, but also their comorbidities such as diabetes mellitus (75%), dyslipidaemia (75%), and obesity (59.4%). The presence of comorbidities resulted in a high percentage of patients with polypharmacy (87.5%); they took about 8.0 (SD of 2.3) different medicines per day and from 6 to 24 pills/day ( Table 1 ). Regarding the number of Supermed used by the patients, the mean was 4 (SD of 1.1) devices per participant. The most used periods were: “after breakfast”, “after lunch” and “after dinner”.

**Table 1:** Baseline characteristics of the hypertensive older adults followed at the Primary Care Unit

Variable		n (%)	Median (range)
Age, years		32	70.5 (62-84)
Gender			
	Female	21 (65.6)	
	Male	11 (34.4)	
Ethnicity			
	White	23 (71.9)	
	Black	7 (21.9)	
	Mixed	2 (6.3)	
Education, years of schooling			3.5 (0-11)
	Illiterate (no school year)	6 (18.8)	
	1-4	19 (59.4)	
	5 or more	7 (21.9)	
Marital status			
	Married	21 (65.6)	
	Widower	10 (31.3)	
	Single	1 (3.1)	
Family income, minimum wage			2.5 (1-6)
	≤1	6 (18.8)	
	2-3	14 (43.8)	
	4-6	12 (37.5)	
Occupation			
	Retired	28 (87.5)	
	Housekeeper	3 (9.4)	
	Retired but working	1 (3.1)	
Time of continuous drug use (years)		32	17.5 (5-35)
Hypertension diagnosis (years)		32	17.5 (2-40)
Number of comorbidities		32	3.0 (0-4)
Number of medicines taken per day		32	8.0 (5-14)
Number of tablets taken per day		32	11.0 (6-24)

Regarding medication adherence, 81.2% of 32 patients were “less adherent” in the pre-intervention period and 96.9% were “more adherent” in the post-intervention period. It means that the status of 78.1% of patients changed from “less adherent” to “more adherent” after the intervention with Supermed. This change was statistically significant, with p-value<0.001.

In the pre-intervention period, 84.4% of 32 patients reported that they forgot to take their medicines, and 87.5% were careless about the time to take them. In the post-intervention period, only 3,1% reported that they forgot to take their medicines, and 31,2% were careless about the time to take them. The change in answers to the two questions of the Morisky scale^(^^13^^)^ test was statistically significant, with p<0.001.

We compared the patient's blood pressure in the pre- and post-intervention periods and on the day of intervention with Supermed, which represents the time “just before” the intervention. The blood pressures measured in the pre-intervention period and on the intervention day were similar.

The mean SBP and DBP differences between intervention day and post-intervention were 18.5mmHg (p<0.0001) and 4.3mmHg (p<0.007), respectively. The differences between mean SBP and DBP between pre-intervention and post-intervention were 21.6mmHg (p<0.001) and 4.7mmHg (p<0.001), respectively ( Table 2 ).

**Table 2:** Clinical parameters of hypertensive elderly people, before and after the Electronic System for Personalized and Controlled Use of Medicines

Parameter	Time	Mean	Median (range)	Comparison	Difference	95%CI	p value *
Systolic blood pressure (mmHg)	Pre-intervention	151.9	149.0 (100-201)	Pre *versus* post	-21.6	19.42-23.72	<0.0001
	Day of intervention	148.8	146.0 (128-187)	Day of intervention *versus* post	-18.5	14.75-22.20	<0.0001
	Post-intervention	130.3	132.0 (89-182)				
Diastolic blood pressure (mmHg)	Pre-intervention	78.8	79.0 (49-118)	Pre *versus* post	-4.7	3.28-6.16	<0.0001
	Day of intervention	78.4	80.5 (52-96)	Day of intervention *versus* post	-4.3	1.83-6.82	0.0007
	Post-intervention	74.1	74.0 (50-99)				

* p-value was determined using analysis of variance and *t* -based orthogonal contrasts.

95%CI: 95% confidence interval.

The qualitative satisfaction survey performed at the end of the study period revealed that most of the patients liked Supermed, because it helped them to organize their medications, and the alarm clocks reminded them about the time to take the medicines.

“Excellent! I think it is excellent because it really organizes. I was very disorganized with my medicines. I did not have an appropriate place and placed them anywhere. Sometimes, when I was in a hurry, and I was late to take a medicine, I made a great confusion. In the boxes, they are organized; it is just to take the box. It is great, wonderful!” (Patient 5)

Some patients, in particular those with hearing problems, complained the alarm volume was too low and they could not hear it. In these situations, some family members heard the alarm and warned the patients, helping them to improve medication adherence and demonstrating that the family provided care to them.

“It is fantastic because I sometimes forget the time to take. But the alarm clock does not let me forget. Even my husband, he is now following the right schedule!” (Patient 15)

The patients reported that the instructions provided by the health professionals at the Primary Care Unit, as well as the labels identifying the time to take the medicines, helped to guide and remind them about the exact time or period of the day that they should take a given medication.

“Oh! It's smart, very smart. Because even if we want to make confusion, it guides you. Because the picture is… a guide. It really shows… the intake time: day, night, everything is organized. Good, good, good, good!” (Patient 21)

Most patients reported that their general health conditions improved in the last few months after the intervention with Supermed; in particular, their blood pressure did not rise anymore.

“Oh, it improved a lot! Before, I didn't take all the pills. Now, with the boxes, I don't forget anything!” (Patient 12)

“Oh, it improved a lot because I was very careless, I didn't take the medicines correctly, skipped the time, forgot; when I remembered it was late. The blood pressure was always raised! I have told you! It was up to 17, 18, 20. Now it's no more than 13 by 7, 12, sometimes it is too low!” (Patient 22)

## DISCUSSION

The study showed that Supermed as reminder device improved medication adherence and markedly contributed to the reduction in SBP and DBP in patients treated with polypharmacy. In Brazil, there are no data analysing electronic medication devices as a tool for improving medication adherence in hypertensive older adults. In this regard, to the best of our knowledge this study is the first to demonstrate that this technology, mainly in older adults, could be useful in the management of hypertension. Strategies such as Supermed, which remind patients about drug intake at the appropriate frequency, are attractive concepts for clinical practice in Primary Health Care.

Some studies using a blister-calendar pack as reminder for hypertensive diabetic patients have reported similar results.^(^^9^^,^^15^^)^ Hence, the use of Supermed seems to be a good strategy to increase medication adherence in older adults, who, sometimes, do not take medicines correctly due to forgetfulness and/or functional limitations, such as difficulty to open the packs, read the labels, and understand the healthcare professionals’ instructions.^(^^16^^,^^17^^)^ The World Health Organization (WHO) highlights that inappropriate adherence to long-term therapies is a serious problem worldwide, and that the mean adherence rate in developed countries is 50%, while in developing countries the rates are lower.^(^^18^^)^

To design specific and effective interventions, it is essential to identify the specific individual barriers that affect the older adults’ adherence to medications.^(^^19^^,^^20^^)^ Pill intake forgetfulness was a main reason for inadequate adherence to antihypertensive therapy.^(^^3^^,^^5^^)^ Before the intervention, forgetfulness was frequent among the older adults included in our study, but decreased significantly, with Supermed showing its value in improving adherence. A study with 8,692 hypertensive patients, with a mean age of 63.4 years, showed non-adherence to medication was caused mainly by forgetfulness (60.8%), being busy (18.5%), and other reasons (8.1%), including travel, hospitalization, and inability to go to the pharmacy. Hence, the health professionals should analyze the patient's daily routine to develop a suitable medication schedule, as well as to define the best strategies to improve medication adherence.^(^^19^^,^^20^^)^

In this sense, Supermed was designed to organize the medicines into single-dose compartments, labelled with the day and time to take the medicine. The labels also contained a braille description of the information to enable the visually disabled to use this device.^(^^10^^)^ Our results are in line with a previous study that reports that a healthcare program consisting of individual instructions about medicines, organization of medicines in packs labelled with the intake time, and regular following by clinical pharmacists, has increased medication adherence in older patients from 61.2% to 96.9%, and improved blood pressure control.^(^^21^^)^

The Supermed with the alarm is a relatively cheap device, currently costing about US$30 and can be accessible to the population. In addition, it can be equipped with an electronic system composed of a power source, an embedded system composed of a memory card, and software to control the alarm clock and record the box opening. These accessories aid the health professionals in assessing whether the patients were taking the medicines correctly, but increases the cost of Supermed by nearly fourfold.^(^^10^^)^ Due to their high cost, electronic pillboxes that record every opening event have been used primarily as research tools.^(^^22^^)^ The large-scale production and marketing of the developed prototypes can markedly reduce the final cost, making them accessible to the low-income population.^(^^10^^)^ Compared with self-report questionnaires, electronic monitoring of drug intake can mitigate the patients’ over-reporting of adherence.^(^^22^^,^^23^^)^

In addition to technology strategies, the need for a multidisciplinary approach towards poor adherence to medication intake is being widely stressed and encouraged. In this approach, pharmacists play a vital role in the healthcare system and can substantially contribute to improve adherence and the value of pharmacotherapy,^(^^4^^,^^24^^)^ as well as to incorporate medication habits into the patients’ daily schedule and routine.^(^^25^^)^ The WHO concluded improving patients’ adherence may have a greater effect on health than any other improvement in therapy.^(^^18^^)^

From the reports obtained in the interviews, the device facilitated the self-management of pharmacotherapy, and gave a feeling of greater autonomy and independence to the older adults, which contributes to a better perception regarding themselves and their quality of life in the aging process.^(^^26^^,^^27^^)^

The main limitations of the present study were that it was conducted in only one Primary Care Unit, with a small sample, and for a short time, which prevent generalizations to all older adults and an in-depth assessment of the patient's acceptance. The influence of the Hawthorne effect (the phenomenon by which individuals change their behaviour due to participating in a study)^(^^28^^)^ may have led the patients to take their medications more regularly than they usually did. In addition, the fact that we did not examine the older adult's cognition, and non-adherent patients being less likely volunteer for the study, could have introduced bias in the measures of adherence. Our data support the need for more robust studies in Brazilian primary care, with more older adults and complementary measures of medication adherence.

The Supermed will also go through several stages of improvement. Among the foci of future work are the development of a circuit of its own, with the definition of components necessary for the compilation of the electronic system to replace the Arduino, and miniaturization of the box to produce a more pleasant visual presentation. Alarm volume control settings can also be added to the system. Another idea is the development of a digital bracelet as an optional element of the system.

Device connectivity is also an improvement to be considered. A Wi-Fi module added to the device will allow monitoring of its use in real time. Therefore, healthcare professionals may check information generated from Supermed's embedded system, through a smartphone app or website. Apps are a new resource to treat non-adherence problems,^(^^29^^)^ allowing to potentiate solutions presented in this article.

This preliminary study demonstrated that it is possible to develop new strategies to organize medicines for the care of older patients, which provide satisfactory results and improve efficiency of treatment.

## CONCLUSION

Together, the patients’ reports evidenced their satisfaction with the use of the Electronic System for Personal and Controlled Use of Medications, especially with respect to organization and forgetfulness. They felt safer to take their medicines, which improved their medication adherence, and consequently their blood pressure control. Organization of the medications in sachets, labelled with the day and time to take the medicine, also helped patients to carry the medicines when they need to leave their homes; it promoted self-care and autonomy for the older adults, who started to actively participate in their own health treatments. Hence, the use of Electronic System for Personal and Controlled Use of Medications significantly improved self-reported medication adherence and control of blood pressure in a hypertensive older adult population with polypharmacy.
