# Supplementary material for: The use of an electronic medication organizer device with alarm to improve medication adherence of older adults with hypertension
Source: Einstein (Sao Paulo). 2021 Sep 13;19:eAO6011. doi: 10.31744/einstein_journal/2021AO6011 (PMC8439558; doi:10.31744/einstein_journal/2021AO6011)
Supplement: Translation

# Translation: Uso de um dispositivo eletrônico organizador de medicamentos com alarme para melhorar a adesão medicamentosa de idosos com hipertensão

**Authors:** Liliana Batista Vieira, Adriano Max Moreira Reis, Celso de Ávila Ramos, Tiago Marques dos Reis, Silvia Helena de Bortoli Cassiani

**Affiliations:** 1 Ribeirão PretoSP Brasil Escola de Enfermagem de Ribeirão Preto, Universidade de São Paulo, Ribeirão Preto, SP, Brasil.; 2 Belo HorizonteMG Brasil Faculdade de Farmácia, Universidade Federal de Minas Gerais, Belo Horizonte, MG, Brasil.; 3 AlfenasMG Brasil Universidade José do Rosário Vellano, Alfenas, MG, Brasil.; 4 AlfenasMG Brasil Universidade Federal de Alfenas, Alfenas, MG, Brasil.

**Keywords:** Uso de medicamentos, Equipamentos e provisões, Sistemas de alerta, Idoso, Segurança do paciente

## Abstract

**Objetivo:**

Avaliar se o uso de um dispositivo organizador de medicamentos eletrônico, mensal, equipado com um despertador, chamado Sistema Eletrônico para Uso Personalizado e Controlado de Medicamentos (Supermed), melhora a adesão à medicação de idosos com hipertensão.

**Métodos:**

Trata-se de um estudo qualiquantitativo, prospectivo, antes e depois, realizado com 32 pacientes idosos, com diagnóstico de hipertensão, recrutados em uma Unidade Básica de Saúde do Brasil.

**Resultados:**

Os principais desfechos foram a melhor adesão à medicação e o controle da pressão arterial após a intervenção com Supermed. Quanto à adesão medicamentosa, 81,2% dos pacientes eram “menos aderentes” no período pré-intervenção, e 96,9% eram “mais aderentes” no período pós-intervenção. Isso significa que 78,1% dos pacientes mudaram de “menos aderentes” para “mais aderentes” após a intervenção com Supermed (p<0,001). As diferenças da pressão arterial sistólica e diastólica média entre o dia da intervenção e pós-intervenção foram 18,5mmHg (p<0,0001) e 4,3mmHg (p<0,007), respectivamente, e as diferenças entre as médias da pressões arteriais sistólica e diastólica entre os períodos pré-intervenção e pós-intervenção foram de 21,6mmHg (p<0,001) e 4,7mmHg (p<0,001), respectivamente.

**Conclusão:**

O uso de Supermed melhorou de forma significativa a adesão medicamentosa e o controle da pressão arterial em uma população idosa hipertensa.

## INTRODUÇÃO

Hipertensão é um dos principais fatores de risco modificáveis para eventos cardiovasculares e mortalidade em idosos.^(^^1^^)^Apesar da disponibilidade de tratamento efetivo com medicamentos anti-hipertensivos, a falta de adesão à terapêutica pode comprometer o controle da pressão arterial,^(^^1^^–^^4^^)^e o uso de tratamento medicamentoso baseado em evidências diminui a morbimortalidade cardiovascular.^(^^1^^,^^2^^)^

A adesão ao tratamento medicamentoso é um fenômeno psicocomportamental complexo e individual. Trata-se de um problema crescente entre os idosos, influenciado por fatores associados ao médico, ao tratamento específico, ao sistema de saúde, a comorbidades, à compreensão do paciente sobre sua doença clínica e à satisfação do paciente com o tratamento.^(^^4^^,^^5^^)^Essa diversidade de fatores pode levar à não adesão intencional ou não do paciente.^(^^4^^)^A não adesão intencional é determinada pela decisão ativa do paciente de não tomar a medicação conforme prescrito. Por outro lado, a não adesão não intencional resulta de outros fatores, como esquecimento, não entendimento do esquema terapêutico e falta de acesso à medicação.^(^^4^^,^^6^^)^Estratégias que lembrem os pacientes de tomar os medicamentos são importantes para melhorar a adesão.^(^^3^^)^

Os farmacêuticos são alguns dos profissionais mais importantes para promover o uso racional de medicamentos, pois garantem que os esquemas terapêuticos sejam seguros e efetivos. As estratégias que os farmacêuticos clínicos podem adotar para melhorar a adesão aos medicamentos incluem fornecer instruções aos pacientes sobre sua doença, tratamento e estilo de vida; monitorar e registrar a pressão arterial; fornecer lembretes dos medicamentos, incluindo telefonemas e dispor de sistemas de dispensação, como monitoramento eletrônico, cartelas ou porta comprimidos para organizar doses diárias.^(^^7^^–^^9^^)^No Brasil, existem alguns dispensadores de medicamentos para o dia ou para a semana, equipados com despertadores, mas não um dispositivo organizador eletrônico mensal.

Foi desenvolvido um dispositivo mensal de medicação personalizado, denominado Sistema Eletrônico de Uso Personalizado e Controlado de Medicamentos, ou Supermed.^(^^10^^)^O dispositivo é equipado com um despertador e ajuda o idoso a organizar e gerenciar seu uso diário de medicamentos por 1 mês, de forma prática e fácil, além de auxiliar os profissionais da saúde a avaliar se os pacientes estão tomando os medicamentos corretamente. O Supermed oferece aos idosos – geralmente não familiarizados com tecnologia moderna – instruções claras, simples e diretas; seu uso requer o mínimo de esforço físico e conhecimento sobre dispositivos eletrônicos.^(^^10^^)^A hipótese testada no estudo foi a de que o uso do Supermed pode aumentar a adesão do paciente ao tratamento medicamentoso.

## OBJETIVO

Avaliar se o uso do Sistema Eletrônico de Uso Personalizado e Controlado de Medicamentos melhora a adesão de idosos hipertensos ao uso de medicamentos.

## MÉTODOS

### Delineamento e local do estudo

A pesquisa foi realizada com duas abordagens metodológicas complementares, quantitativa e qualitativa, para maior compreensão dos fenômenos estudados. A etapa quantitativa foi realizada com um desenho quase-experimental, prospectivo, antes e depois. Por sua vez, a qualitativa foi feita com análise de conteúdo temático. Ambas foram desenvolvidas em uma Unidade Básica de Saúde de Ribeirão Preto (SP).

### População

A amostra de conveniência foi formada por 32 pacientes idosos, considerando os critérios de seleção e o cronograma da pesquisa. Os critérios de inclusão foram idade ≥60 anos; ambos os sexos; falar português; tomar diariamente cinco ou mais medicamentos sólidos, incluindo pelo menos um anti-hipertensivo; ter pressão arterial sistólica (PAS) ≥140mmHg ou PAS ≥130mmHg, se hipertenso e diabético e fazer acompanhamento regular com um médico da Unidade Básica de Saúde. Os valores da PAS foram definidos de acordo com as Diretrizes Brasileiras de Hipertensão.^(^^11^^)^Os critérios de exclusão foram idoso diagnosticado com transtornos mentais ou comprometimento cognitivo. O critério de inclusão de cinco ou mais medicamentos tinha o objetivo de garantir a participação dos idosos com polifarmácia intensa (mais que cinco medicamentos por paciente).^(^^12^^)^

### Aprovação ética

Todos os idosos que aceitaram participar deste estudo assinaram o Termo de Consentimento Livre e Informado. Todos os procedimentos realizados em estudos envolvendo participantes humanos obedeceram aos padrões éticos do Comitê de Ética em Pesquisa da instituição e da declaração de Helsinque de 1964 e suas alterações posteriores, ou padrões éticos comparáveis (Comitê de Ética em Pesquisa da Universidade de São Paulo, Ribeirão Preto, São Paulo, Colégio Brasileiro de Enfermagem, protocolo 1.398/2011).

### Intervenção

O Supermed foi utilizado como dispositivo de lembrete de medicamentos. Ele é composto de um dispositivo organizador de medicamentos, compartimentos plásticos para colocar doses individuais, etiquetas de instrução, um despertador e um sistema eletrônico, que controla o despertador e registra a abertura da caixa ( Figura 1 ).^(^^10^^)^

**Figura 1:** Sistema Eletrônico de Uso Personalizado e Controlado de Medicamentos, um dispositivo organizador de medicamentos eletrônico, mensal, com despertador. A) Dispositivo organizador de medicamentos identificado com o horário correto de tomada, e preenchido com sachês contendo doses únicas dos medicamentos prescritos, em quantidades suficientes para 1 mês de tratamento. Os nomes dos medicamentos são escritos em um rótulo na parte de trás de cada dispositivo; B) Cada sachê contendo dose única é identificado com data e horário da tomada; C) Detalhes dos rótulos: “tomar depois do café da manhã”; D) Alarme programado para soar no horário combinado com o idoso

O Supermed foi projetado para lembrar o paciente do horário para tomar a medicação; verificar se o paciente desligou o despertador e abriu a caixa para tomar a medicação, bem como registrar os horários em que o dispositivo foi aberto, o que pressupõe a tomada dos medicamentos. O protótipo do Supermed inclui uma gaveta com capacidade para 1 mês de suprimento de medicamentos, timers sonoros e visuais e um cartão de memória para registrar os horários em que a caixa foi aberta/fechada (de forma programada e não programada). Essa informação é posteriormente transferida para um computador. A prototipagem evolutiva foi usada para desenvolver o Supermed por meio da plataforma Arduino e da programação C. Para ler dados de alarme e abertura/fechamento da caixa, o programa foi desenvolvido na linguagem Java.^(^^10^^)^

O número total de dispositivos utilizados variou entre os pacientes. Cada Supermed tinha uma cor diferente e estava preenchido com compartimentos (sachês) contendo doses únicas dos medicamentos prescritos. Os idosos receberam dispositivos identificados com o horário de tomada do medicamento, por exemplo: “depois de acordar”, “antes do café da manhã”, “depois do café da manhã”, “antes do almoço”, “depois do almoço”, “antes do jantar”, “depois do jantar” e/ou “na hora de dormir”. Os nomes dos medicamentos contidos em cada caixa foram escritos em um rótulo na parte de trás, e o número total de compartimentos foi suficiente para 1 mês de tratamento. As caixas foram preparadas a cada mês na presença do paciente, como uma oportunidade para esclarecer suas dúvidas e melhorar o entendimento sobre sua doença clínica, das instruções de medicação e da importância da adesão ao tratamento. Esse encontro durou de 1 a 2 horas para cada paciente. Os pacientes receberam uma sacola para transportar as caixas e, após o término do período de estudo, puderam ficar com os dispositivos.

O Supermed foi introduzido na Unidade Básica de Saúde em fevereiro de 2013. O período de pré-intervenção continha dados de pacientes de março de 2012 a janeiro de 2013, e, no período pós-intervenção, os dados foram coletados dos mesmos pacientes entre fevereiro e agosto de 2013. Este trabalho apresenta testes e resultados da funcionalidade do alarme do dispositivo. Outras funcionalidades do Supermed, como o registro dos horários, serão apresentadas futuramente.

### Resultados e medidas

#### Pressão arterial

Foi medida conforme recomendado pelas Diretrizes Brasileiras de Hipertensão,^(^^11^^)^utilizando um monitor de pressão arterial digital calibrado e validado (modelo Omron HEM-742INT). Cada medição foi realizada três vezes, e o valor médio das duas últimas medições foi calculado. As medidas da PAS e da pressão arterial diastólica (PAD) foram realizadas nos seguintes momentos:

– Período de pré-intervenção: antes da introdução do Supermed, foram realizados cinco encontros com cada paciente, de março de 2012 a janeiro de 2013.
– Dia da intervenção: durante o mês de fevereiro de 2013, no dia da introdução do Supermed, foi realizado um encontro com o paciente.
– Período pós-intervenção: após a introdução do Supermed, foram realizados cinco encontros (um encontro por mês) com cada paciente, de março de 2013 a agosto de 2013.

#### Adesão aos medicamentos

Foi determinada utilizando-se a Escala de Adesão Terapêutica de Morisky,^(^^13^^)^composta de quatro perguntas, para analisar os comportamentos habituais dos pacientes em relação à tomada dos medicamentos, nos períodos pré e pós-intervenção. As respostas foram pontuadas como sim (zero) e não (um); os pacientes com escores finais de zero a dois e três a quatro foram considerados como “menos aderentes” e “mais aderentes”, respectivamente. O teste foi aplicado antes e depois da intervenção.

#### Pesquisa de satisfação

Ao final do período de estudo (agosto de 2013), foi realizada uma entrevista semiestruturada com cada paciente, utilizando quatro perguntas norteadoras: O que você pensa das caixas organizadoras de medicamentos? O que você acha do despertador? O que você acha das instruções de uso do Supermed, como os rótulos ilustrados? Como você avalia sua saúde geral nos últimos meses? As entrevistas foram gravadas e totalmente transcritas. Os dados foram analisados por meio de análise de conteúdo temático, conforme o estilo de análise editorial. A análise de conteúdo tem o objetivo de desconstruir a fala para ver “o que está por trás do conteúdo manifestado”.^(^^14^^)^

### Análise de dados

Os dados foram analisados utilizando-se o software estatístico SAS^®^9.0. As variáveis de interesse (PAS e PAD) nos três períodos de coleta de dados (pré-intervenção, dia da intervenção e pós-intervenção) foram comparadas por meio da análise de variância (Anova) e contrastes ortogonais baseados em *t* . As variáveis da escala de Morisky,^(^^13^^)^obtidas antes e depois da intervenção, foram comparadas utilizando-se o teste de McNemar. Os valores de p<0,05 foram considerados significativos.

## RESULTADOS

Este estudo incluiu 32 pacientes idosos (21 mulheres), com média de idade de 71,4 anos, com desvio-padrão (DP) de 5,6 anos. A maioria era aposentada (87,5%) e de baixa renda – abaixo de três salários mínimos – (62,6%) e baixa escolaridade – analfabeto ou menos de 4 anos de estudo – (78,2%). Os pacientes tinham sido diagnosticados com hipertensão por média de 19,4 anos antes da entrada no estudo e vinham tomando medicamentos de longo prazo desde então para tratar não apenas a hipertensão, mas também suas comorbidades, como *diabetes mellitus* (75%), dislipidemia (75%) e obesidade (59,4%). A presença de comorbidades resultou em alto percentual de pacientes com polifarmácia (87,5%); eles tomavam cerca de 8,0 (DP de 2,3) medicamentos diferentes ao dia e de seis a 24 comprimidos ao dia ( Tabela 1 ). Em relação ao número de dispositivos Supermed utilizados pelos pacientes, a média foi de 4 (DP de 1,1) dispositivos por participante. Os períodos mais utilizados foram: “depois do café da manhã”, “depois do almoço” e “depois do jantar”.

**Tabela 1:** Características iniciais de idosos hipertensos acompanhados em uma Unidade Básica de Saúde

Variável		n (%)	Mediana (variação)
Idade, anos		32	70,5 (62-84)
Sexo			
	Mulheres	21 (65,6)	
	Homens	11 (34,4)	
Etnia			
	Brancos	23 (71,9)	
	Negros	7 (21,9)	
	Mistos	2 (6,3)	
Escolaridade, anos de estudo			3,5 (0-11)
	Analfabetos (nenhum)	6 (18,8)	
	1-4	19 (59,4)	
	5 ou mais	7 (21,9)	
Estado civil			
	Casado	21 (65,6)	
	Viúvo	10 (31,3)	
	Solteiro	1 (3,1)	
Renda familiar, salário mínimo			2,5 (1-6)
	≤1	6 (18,8)	
	2-3	14 (43,8)	
	4-6	12 (37,5)	
Ocupação			
	Aposentado	28 (87,5)	
	Dona de casa	3 (9,4)	
	Aposentado, mas trabalhando	1 (3,1)	
Tempo de uso contínuo de medicamento, anos		32	17,5 (5-35)
Diagnóstico de hipertensão, anos		32	17,5 (2-40)
Número de comorbidades		32	3,0 (0-4)
Número de medicamentos por dia		32	8,0 (5-14)
Número de comprimidos por dia		32	11,0 (6-24)

Quanto à adesão aos medicamentos, 81,2% dos 32 pacientes foram “menos aderentes” no período pré-intervenção, e 96,9% foram “mais aderentes” no período pós-intervenção. Isso significa que a condição de 78,1% dos pacientes passou de “menos aderente” para “mais aderente” após a intervenção com Supermed. Essa mudança foi estatisticamente significativa, com valor de p<0,001.

No período pré-intervenção, 84,4% dos 32 pacientes relataram que esqueciam de tomar seus medicamentos, e 87,5 % eram negligentes quanto ao horário de tomada. No período pós-intervenção, somente 3,1% relataram ter esquecido de tomar seus medicamentos, e 31,2% eram descuidados quanto ao horário para tomá-los. A mudança nas respostas às duas perguntas da escala de Morisky^(^^13^^)^foi estatisticamente significativas, com p<0,001.

Comparamos a pressão arterial dos pacientes nos períodos pré- e pós-intervenção e no dia da intervenção com o Supermed, o que representa o momento “logo antes” da intervenção. As pressões sanguíneas medidas no período pré-intervenção e no dia da intervenção foram semelhantes.

As diferenças médias de PAS e PAD entre o dia da intervenção e no pós-intervenção foram 18,5mmHg (p<0,0001) e 4,3mmHg (p<0,007), respectivamente. As diferenças entre PAS e PAD médias entre pré-intervenção e pós-intervenção foram 21,6mmHg (p<0,001) e 4,7mmHg (p<0,001), respectivamente ( Tabela 2 ).

**Tabela 2:** Parâmetros clínicos de idosos hipertensos, antes e depois do Sistema Eletrônico para Uso Personalizado e Controlado de Medicamentos

Parâmetro	Momento	Média	Mediana (variação)	Comparação	Diferença	IC95%	Valor de p *
Pressão arterial sistólica (mmHg)	Pré-intervenção	151,9	149,0 (100-201)	Pré *versus* pós	-21,6	19,42-23,72	<0,0001
	Dia da intervenção	148,8	146,0 (128-187)	Dia da intervenção *versus* pós	-18,5	14,75-22,20	<0,0001
	Pós-intervenção	130,3	132,0 (89-182)				
Pressão diastólica (mmHg)	Pré-intervenção	78,8	79,0 (49-118)	Pré *versus* pós	-4,7	3,28-6,16	<0,0001
	Dia da intervenção	78,4	80,5 (52-96)	Dia da intervenção *versus* pós	-4,3	1,83-6,82	0,0007
	Pós-intervenção	74,1	74,0 (50-99)				

* Valor de p determinado por análise de variância e contraste ortogonal baseado em *t* .

IC95%: intervalo de confiança de 95%.

A pesquisa de satisfação qualitativa realizada ao final do período de estudo revelou que a maioria dos pacientes gostou do Supermed, porque os ajudava a organizar seus medicamentos, e os despertadores os lembravam da hora de tomar os medicamentos.

“Excelente! Eu acho que é excelente porque ele realmente organiza. Estava muito desorganizado com meus remédios. Não tinha um lugar certo e colocava em qualquer lugar. Às vezes, quando estava com pressa, e tomava um remédio atrasado, fazia uma grande confusão. Nas caixas eles ficam organizados; é só levar a caixa. É ótimo, maravilhoso!” (Paciente 5)

Alguns pacientes, em particular aqueles com problemas de audição, reclamaram que o volume do alarme era muito baixo e que não conseguiam ouvir. Nessas situações, alguém da família ouvia o alarme e alertava o paciente, ajudando a melhorar a adesão aos medicamentos e demonstrando que a família dava assistência.

“É fantástico porque às vezes esqueço o horário de tomar. Mas o alarme não me deixa esquecer. Até meu marido agora está seguindo o regime certo!” (Paciente 15)

Os pacientes relataram que as instruções fornecidas pelos profissionais da saúde da Unidade Básica de Saúde, assim como os rótulos que identificavam o horário de tomada dos medicamentos, ajudaram a orientar e a lembrá-los do horário ou do período exato do dia em que deveriam tomar um determinado medicamento.

“Ah! É inteligente, muito inteligente. Porque mesmo que a gente queira fazer confusão, ele te orienta. Porque a ilustração é… um guia. Mostra mesmo… o horário de tomar: dia, noite, tudo é organizado. Bom, bom, bom, bom!” (Paciente 21)

A maioria dos pacientes relatou que suas condições gerais de saúde melhoraram nos últimos meses após a intervenção com o Supermed; em particular, a pressão arterial não subiu mais.

“Ah, melhorou muito! Antes, eu não tomava todos os remédios. Agora, com as caixas, eu não esqueço nada!” (Paciente 12)

“Ah, melhorou muito porque eu era muito descuidado, não tomava os remédios direito, esquecia o horário, esquecia; quando lembrava era tarde. A pressão sempre estava alta! Eu já te contei! Subia a 17, 18, 20. Agora não passa de 17 por 7, 12, às vezes fica baixa demais!” (Paciente 22)

## DISCUSSÃO

O estudo mostrou que o Supermed, como dispositivo de lembrete, melhorou a adesão aos medicamentos e contribuiu, de forma importante, para a redução da PAS e da PAD em pacientes tratados com polifarmácia. No Brasil, não há dados que analisem dispensadores eletrônicos de medicamentos como ferramenta para melhorar a adesão de idosos hipertensos à terapêutica. Nesse sentido, até onde sabemos, este estudo é o primeiro a demonstrar que essa tecnologia, principalmente em idosos, poderia ser útil no manejo da hipertensão. Estratégias como o Supermed, que lembrem os pacientes sobre o uso dos medicamentos com a frequência adequada, são conceitos atraentes para a prática clínica na Atenção Primária à Saúde.

Alguns estudos usando uma embalagem com cartelas-calendário como lembrete para pacientes diabéticos hipertensos relataram resultados semelhantes.^(^^9^^,^^15^^)^Dessa forma, o uso do Supermed parece ser uma boa estratégia para aumentar a adesão de idosos, que, às vezes, não tomam medicamentos corretamente por esquecimento e/ou limitações funcionais, como dificuldade para abrir as embalagens, ler os rótulos e entender as instruções dos profissionais da saúde.^(^^16^^,^^17^^)^A Organização Mundial da Saúde (OMS) destaca que a adesão inadequada aos tratamentos de longo prazo é um problema sério em todo o mundo, e a taxa média de adesão nos países desenvolvidos é de 50%, enquanto nos países em desenvolvimento as taxas são mais baixas.^(^^18^^)^

Para elaborar intervenções específicas e eficazes, é essencial identificar as barreiras individuais que afetam a adesão dos idosos aos medicamentos.^(^^19^^,^^20^^)^Esquecer de tomar o medicamento foi a principal razão para a adesão inadequada à terapia anti-hiperpertensiva.^(^^3^^,^^5^^)^Antes da intervenção, o esquecimento era frequente entre os idosos incluídos neste estudo, mas diminuiu significativamente com o Supermed, mostrando seu valor na melhora da adesão. Um estudo com 8.692 pacientes hipertensos, com idade média de 63,4 anos, demonstrou que a não adesão à medicação foi causada principalmente por esquecimento (60,8%), estar ocupado (18,5%) e outras razões (8,1%), incluindo viagens, hospitalização e incapacidade de ir à farmácia. Assim, os profissionais da saúde devem analisar a rotina diária do paciente para desenvolver um regime adequado de medicamentos, bem como definir as melhores estratégias para melhorar a adesão aos medicamentos.^(^^19^^,^^20^^)^

Nesse sentido, o Supermed foi projetado para organizar os medicamentos em compartimentos de dose única, rotulados com o dia e o horário para tomá-los. Os rótulos também continham uma descrição em braile das informações, para permitir que os deficientes visuais usem esse dispositivo.^(^^10^^)^Nossos resultados estão alinhados com um estudo anterior que relata que um programa de saúde, composto de instruções individuais sobre medicamentos, a organização deles em embalagens rotuladas com o horário de tomada e o acompanhamento regular por farmacêuticos clínicos, aumentou de 61,2% para 96,9% a adesão de idosos aos medicamentos e melhorou o controle da pressão arterial.^(^^21^^)^

O Supermed com o alarme é um dispositivo relativamente barato, atualmente custando cerca de US$30, e pode ser acessível para a população. Além disso, pode ser equipado com um sistema eletrônico composto de uma fonte de energia, um sistema incorporado composto por um cartão de memória e software, para controlar o alarme e registrar a abertura da caixa. Esses acessórios auxiliam os profissionais da saúde a avaliar se os pacientes estavam tomando os medicamentos corretamente, mas aumentam o custo do Supermed em quase quatro vezes.^(^^10^^)^Devido ao seu alto custo, os dispensadores eletrônicos que registram cada abertura têm sido usados principalmente como ferramentas de pesquisa.^(^^22^^)^A produção e a comercialização em larga escala dos protótipos desenvolvidos podem reduzir significativamente o custo final, tornando acessível à população de baixa renda.^(^^10^^)^Comparado com questionários autorrelatados, o monitoramento eletrônico do uso de medicamentos pode diminuir o risco de os pacientes hipervalorizarem a adesão.^(^^22^^,^^23^^)^

Além das estratégias tecnológicas, a necessidade de uma abordagem multidisciplinar para a baixa adesão ao uso dos medicamentos está sendo amplamente enfatizada e incentivada. Nessa abordagem, os farmacêuticos desempenham papel vital no sistema de saúde e podem contribuir substancialmente para melhorar a adesão e o valor da farmacoterapia,^(^^4^^,^^24^^)^bem como incorporar os medicamentos como hábitos em seus horários e rotina diários.^(^^25^^)^A OMS conclui que melhorar a adesão dos pacientes pode ter um efeito maior na saúde do que qualquer outra melhora no tratamento.^(^^18^^)^

A partir dos relatos obtidos nas entrevistas, o dispositivo facilitou o autogestão da farmacoterapia e deu uma sensação de maior autonomia e independência aos idosos, o que contribui para uma melhor percepção de si próprios e qualidade de vida no processo de envelhecimento.^(^^26^^,^^27^^)^

As principais limitações deste estudo foram ter sido conduzido em apenas uma Unidade Básica de Saúde, com amostra pequena e por pouco tempo, o que impede a generalização dos resultados para todos os idosos e uma avaliação aprofundada da aceitação pelos pacientes. A influência do efeito Hawthorne (fenômeno pelo qual os indivíduos mudam seu comportamento por estarem participando em um estudo)^(^^28^^)^pode ter levado os pacientes a tomarem seus medicamentos com mais regularidade do que normalmente faziam. Além disso, os fatos de não se ter examinado a cognição dos idosos e de que pacientes não aderentes provavelmente seriam voluntários para o estudo podem ter introduzido viés nas medidas de adesão. Os presentes dados corroboram a necessidade de estudos mais robustos na Atenção Primária brasileira, com mais idosos e medidas complementares de adesão a medicamentos.

O Supermed também passará por diversas etapas de melhoria. Entre os focos do trabalho futuro estão o desenvolvimento de um circuito próprio, com a definição de componentes necessários para a compilação do sistema eletrônico para substituir o Arduino e a miniaturização da caixa para uma apresentação visual mais agradável. Configurações de controle de volume do alarme também podem ser adicionadas ao sistema. Outra ideia é o desenvolvimento de uma pulseira digital como elemento opcional do sistema.

A conectividade do dispositivo também é uma melhoria a ser considerada. Um módulo Wi-Fi adicionado ao dispositivo permitirá o monitoramento de seu uso em tempo real. Assim, os profissionais da saúde podem checar as informações geradas a partir do sistema incorporado ao Supermed, por meio de um aplicativo para smartphone ou site. Os aplicativos são recursos novos para tratar problemas de não adesão,^(^^29^^)^permitindo potencializar soluções apresentadas neste artigo.

Este estudo preliminar demonstrou que é possível desenvolver novas estratégias para organizar medicamentos para o cuidado de pacientes idosos, que proporcionam resultados satisfatórios e melhoram a eficiência do tratamento.

## CONCLUSÃO

Juntos, os relatos dos pacientes evidenciaram sua satisfação com o uso do Sistema Eletrônico de Uso Personalizado e Controlado de Medicamentos, especialmente quanto à organização e ao esquecimento. Eles se sentiram mais seguros para tomar seus medicamentos, o que melhorou a adesão ao tratamento e, consequentemente, o controle da pressão arterial. A organização dos medicamentos em sachês, rotulados com o dia e o horário para tomar o medicamento, também auxiliou os pacientes a levarem os medicamentos quando precisaram sair de casa e promoveu o autocuidado e a autonomia para os idosos, que passaram a participar ativamente de seus próprios tratamentos de saúde. Assim, o uso do Sistema Eletrônico para Uso Personalizado e Controlado de Medicamentos melhorou significativamente a adesão autorrelatada aos medicamentos e o controle da pressão arterial em uma população idosa, hipertensa e com polifarmácia.
